# Recent progress in ferroptosis: inducers and inhibitors

**DOI:** 10.1038/s41420-022-01297-7

**Published:** 2022-12-29

**Authors:** Yunxi Du, Zhong Guo

**Affiliations:** 1 Center for Biological Science and Technology, Guangdong Zhuhai-Macao Joint Biotech Laboratory, Advanced Institute of Natural Sciences, Beijing Normal University, Zhuhai, China; 2 Key Laboratory of Cell Proliferation and Regulation Biology of Ministry of Education, College of Life Sciences, Beijing Normal University, Beijing, China

**Keywords:** Cancer metabolism, Targeted therapies

## Abstract

Ferroptosis is a new iron-dependent form of programmed cell death characterized by iron accumulation and lipid peroxidation. In recent years, ferroptosis has garnered enormous interest in disease treatment research communities in pursuit to reveal the mechanism and key targets of ferroptosis because ferroptosis is closely related to the pathophysiological processes of many diseases. Recent studies have shown some key targets, such as glutathione peroxidase 4 (GPX4) and System Xc^−^, and several inducers and inhibitors have been developed to regulate these key targets. With the emergence of new ferroptosis targets, studies on inducers and inhibitors have made new developments. The selection and use of inducers and inhibitors are very important for related work. This paper briefly introduces important regulatory targets in the ferroptosis metabolic pathway, lists and categorizes commonly used and recently developed inducers and inhibitors, and discusses their medical application. The paper ends of with potential future research direction for ferroptosis.

## Facts

- Ferroptosis, different from other forms of programmed cell death, is caused by the accumulation of iron and lipid peroxidation.
- Ferroptosis is involved in the occurrence and development of many diseases.
- The application of inducers and inhibitors is crucial in the study of ferroptosis and related diseases.
- Studies on ferroptosis mechanisms and regulators (inducers and inhibitors) promote each other.

## Open questions

- Whether and how to develop more efficient inducers and inhibitors of ferroptosis?
- Whether it is possible to target and regulate ferroptosis through inducers and inhibitors themselves without depending on the carrier?
- The different inducers will change the manifestation of ferroptosis, such as the transmission in cells. Does the regulation of ferroptosis in the body also have this feature?
- How to evaluate the application of inducers that can cause various forms of programmed cell death in the study of ferroptosis?

## Introduction

Ferroptosis, a term coined by Dixon et al. in 2012 [1], is an iron-dependent form of programmed cell death driven by overload of iron and accumulation of lethal lipid peroxidation. Ferroptosis was first observed in 2003 before it was defined by Dixon et al., in cells treated by erastin, a small molecular inducer of ferroptosis found by Dolma et al. [2]. In 2008, Yang et al. identified another compound called RAS synthetic lethal 3 (RSL3), which can activate ferroptosis. They also discovered that cell death caused by RSL3 underwent an iron-dependent, nonapoptotic path [3]. Up to now, extensive research has been conducted on the mechanism of ferroptosis, as summarized below.

It has been reported that excessive iron catalyzes the oxidation of phospholipids having polyunsaturated fatty acids (PUFAs) residues by the Fenton reaction [4]. This process leads to the lipid peroxidation and formation of lipid reactive oxygen species (lipid ROS) and eventually reaches lethal levels of the relevant cells [5]. Apart from the Fenton reaction, the direct induction or the lack of reducing species in cells may also result in the same effect. Eventually, it has been reported that the relevant cells swell before death, which means that the cell membrane is perforated, leading to rupture [6]. In addition, in contrast to other forms of cell death, ferroptosis can distinctively spread through a cell population with a wave-like pattern, and this phenomenon is related to cell leakage [7, 8].

Ferroptosis has become an important research field since it has the potential to be developed into therapies for many diseases by regulated inhibiting or activating of the death process [8, 9]. The regulation of ferroptosis is focused mainly on the regulation of lipid peroxidation level in cells. Although the mechanism and the metabolic pathway of ferroptosis are still poorly understood, multiple types of inhibitors and inducers have been reported in practice [10]. This review intends to summarize the type and mechanism of commonly used and newly discovered ferroptosis inducers and inhibitors based on important metabolic pathways, and discuss their application in scientific research and disease treatment.

### Important regulatory targets in ferroptosis metabolic pathway

Iron ions and ROS driven from lipid and iron metabolism are two key substances involved in ferroptosis. Their associated metabolic pathways have garnered interest from researchers as they play an important role in the mechanism and application of ferroptosis [11].

Acyl-CoA synthetase long chain family member 4 (ACSL4) and voltage-dependent anion channels (VDACs) are known to participate in the production and regulation of ROS in cells. ACSL4 is involved in the synthesis of endogenous PUFAs, turning arachidonic acid into acyl-CoA, which reacts further to produce phospholipid hydroperoxides [12, 13]. Blocking VDACs also leads to the production of ROS. VDACs are a type of channel located on mitochondrial membranes. Ions and some metabolites may cross the membrane through VDACs [14]. In addition to causing changes in ROS level, obstruction of VDACs may also cause mitochondrial damage and apoptosis.

It is also very important for cell viability to prevent the lipid ROS from building up. Glutathione (GSH), a crucial reductant in the regulation of intracellular redox equilibrium, is a tripeptide (L-γ-glutamyl-L-cysteinylglycine) which participates in redox reactions and functions in the metabolism and synthesis of a variety of biological macromolecules [15]. During the process of ferroptosis, GSH is insufficient, such as being previously consumed, thereby being unable to maintain the reducibility of cells.

The production of GSH depends on two enzymes, glutamate–cysteine ligase (GCL, glutamate cysteine synthase) and glutathione synthetase (GSS). Additionally, glutathione relies on glutathione peroxidases (GPXs) to maintain cell reducibility [15]. As a key member of the GPXs family, GPX4 is present in mammals and functions to protect the membrane of cells [16]. Many studies have shown the importance of maintaining an adequate function of the GPX4. Knockout of GPX4 is embryonically lethal [17]. Besides, Yoo et al. demonstrated that adults whose GPX4 were knocked out showed lower drug resistance in a mouse model [18].

System Xc^−^ is located on phospholipid bilayers, where it is known to transport amino acids through the cell membrane. Particularly, cells absorb raw GSH material from the extracellular space by System Xc^−^. As an antitransporter, System Xc^−^ transports cystine and glutamate in and out at a ratio of 1:1 [1]. In view thereof, functional System Xc^−^ is an indispensable part of the process of producing GSH. SLC7A11(xCT) and SLC3A2(4F2hc, CD98) are two subunits of System Xc^−^, which can be targets to regulate ferroptosis. In addition to System Xc^−^, the alanine-serine-cysteine (ASC) preferring amino acid transporter is present in the membrane of some types of cells. The ASC transporter can alternatively transport cysteine across the cell membrane but being unidirectional, and the direction of transport varies among different cells [19]. For cells lacking ASC transporters, such as myeloid-derived suppressor cells, the role of System Xc^−^ in resisting ferroptosis appears to be more important in the absence of its alternative [20].

The GSH metabolic network is not the only network involved in the regulation of ferroptosis. In 2019, ferroptosis suppressor protein 1 (FSP1), formerly named as apoptosis-inducing factor mitochondria-associated 2 (AIFM2), was discovered to regulate ferroptosis independently of GSH [21, 22]. FSP1 acts as an oxidoreductase, using nicotinamide adenine dinucleotide phosphate (NAD(P)H) to change ubiquinone-10 (coenzyme Q10, CoQ10) into CoQH_2_. This mechanism suppresses the propagation of lipid peroxides, thus the expression of FSP1 is connected to ferroptosis resistance in many tumor cell lines [23]. In addition, dihydroorotate dehydrogenase (DHODH) when present in mitochondria can protect them from lipid peroxides by independently reducing ubiquinone to ubiquinol. In this regard, it is important in relevant research to appreciate that DHODH is an essential gene since simply knocking down DHODH may lead to cell death unless where the relevant cells are further treated with uridine. Uridine supplementation can maintain cell proliferation and viability in the absence of the function of DHODH [24]. DHODH was not screened out using clustered regularly interspersed short palindromic repeat (CRISPR)/Cas, probably because of the death of the mutants tested [25, 26].

These metabolic pathways provide many regulatory targets and the space for experimental design for the in-depth study of ferroptosis and in search for therapy. Although there are many ways to induce ferroptosis, the results are notably different. For example, it has been recently reported that the proteasome is affected by inducers in ferroptosis, which is mediated by the transcription factor nuclear factor erythroid-2, like-1 (NFE2L1). The ferroptosis inducers RSL3 and FIN56 increase the activity of NFE2L1, but erastin does not have this effect [27]. Moreover, inhibiting the function of GPX4 directly will not cause the wave-like transmission of death between cell populations, while inhibition of other key targets reportedly showed transmission [6]. The mechanism of this transmission remains unclear to-date, but GPX4 with normal function may be a necessary condition for this phenomenon.

## Ferroptosis inducers

Ferroptosis inducers are indispensable in related studies. New chemical compounds and biomolecules inducing ferroptosis are continuously being found (Table 1). As a first step, choosing an appropriate inducer by category and target may be crucial for relevant research. This choice determines the way in which the selected inducer is obtained and how the study is carried out. For example, some kits based on small molecule inducers and drugs have been developed and commercially available, while some biological macromolecules with induction functions need to be obtained by constructing plasmids.

**Table 1:** Examples of ferroptosis inducers.

Type	Induction mode	Targets	Names	Reference
Small molecular and drug	Target iron metabolism	VDACs	Erastin, RSL5, DIDS	[3, 28–30]
		DMT1	TMZ	[32]
		Ferritin	MMRi62	[33]
	Target lipid metabolism	ACSL4	Sorafenib	[34]
		Cardiolipins	t-BuOOH	[35]
	Target GSH/GPX4 axis	GPX4	RSL3, ML162, DPI7, DPI10, FIN56, Sorafenib	[23, 37, 38, 43–45]
		GCL	BSO	[39]
		System L, System Xc^−^	Erastin, MEII, PE, AE, SAS, Sorafenib	[43–45]
	Target CoQ/FSP1	FSP1	NDP4928	[46]
		SQS	FIN56	[47]
		HMGCR	Statins	[48]
	Target other pathways	DHODH	Brequinar	[24]
		DPEP1	Dexamethasone	[50]
Nanoparticles	Induce the production of iron ions	/	C’ dots, SPION, GA-Fe(II), (UPDA-PEG)@Fe^2+/3+^ nanoparticles	[8, 51, 52, 57]
		Lysosomes	WS_2_ and MoS_2_	[56]
	GSH consumption and Fenton-like reactions	/	CPMNS, LDL-DHA nanoparticles, ChA CQDs, Zinc oxide nanoparticles	[58–60, 62]
	Produce ROS directly	/	PMC	[63]
Nucleic acids and proteins		FSP1	miR-672-3p	[68]
		System Xc^−^	miR-672-3p, IFNγ, p53, BAP1	[71–74]
		TCA cycle	Glutamine	[75, 76]
		ACSL4	PKCβII, miR-129-5p	[77, 91, 92]
		Ferritin	NCOA4	[78]
		Prominin2	miR-129-5p	[91, 92]

Many small molecules and drugs have been proven to cause ferroptosis, and their mechanisms have gradually been understood. As small molecules and drugs are more readily screened than other types of candidates, they are the most abundant type of ferroptosis inducers, which mainly act on the targets as mentioned above (Table 1). According to the targets of inducers, they can be divided into four categories.

### Small molecules and drug inducers targeting iron metabolism

**Fig. 1:**
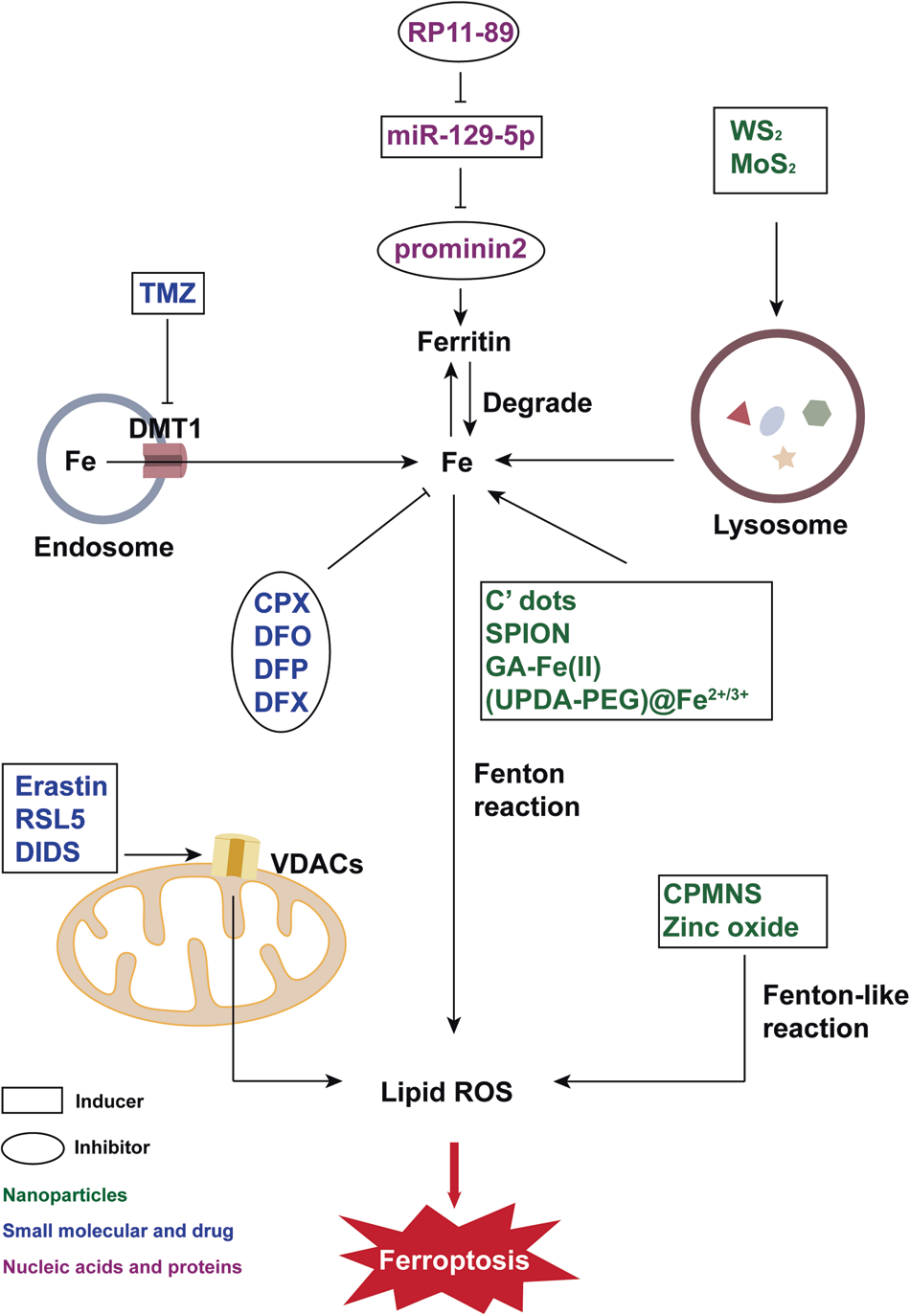
Ferroptosis regulators associated with iron metabolism. Ferroptosis depends on iron ion because it can produce lipid ROS through Fenton reaction. Therefore, substances that regulate iron ions, such as iron chelators and regulators of related proteins or organelles, also function in the regulation of ferroptosis. Additionally, many nanoparticles based on other metal ions are also able to induce ferroptosis by effecting iron ions or directly generating ROS. TMZ temozolomide, DMT1 divalent metal transporter 1, Fe ferrum (iron), CPX ciclopirox olamine, DFO deferoxamine, DFP deferiprone, DFX deferasirox, RSL5 RAS synthetic lethal 5, DIDS 4,4′-diisothiocyanostilbene-2,2′-disulfonic acid, VDACs voltage-dependent anion channels, C’ dots Cornell prime dots, SPION superparamagnetic iron oxide nanoparticles, CPMNS hybrid CoMoO4-phosphomolybdic acid nanosheets, ROS reactive oxygen species.

Erastin, as mentioned above, was first discovered in 2003 as a genotype-selective antitumor agent. Erastin induces ferroptosis in multiple ways, one of which is by targeting VDACs [28]. VDACs have three isoforms, and knockdown of VDAC2 and VDAC3 leads to resistance to erastin in the relevant cells. Erastin would then induce the production of lipid ROS by binding directly with VDAC2. Apart from erastin, RAS synthetic lethal 5 (RSL5), another compound with a similar effect (Fig. 1), binds with VDAC3 to induces ferroptosis [3]. Additionally, VDACs can be inhibited by another small molecular allosteric blocker of mitochondrial anion channels named 4,4′-diisothiocyanostilbene-2,2′-disulfonic acid (DIDS) [29, 30]. Blocking VDACs increases the cellular sensitivity to ionizing radiation and inhibits the repair of DNA damage. Thus, DIDS has been shown to enhance efficacy of radiotherapy in the treatment of cancer [14].

The level of iron in a cell is another important indicator (Fig. 1). Divalent metal transporter 1 (DMT1) is related to the level of intracellular iron and iron homeostasis [31]. A recent study showed that temozolomide (TMZ), an antitumor drug for the treatment of glioblastoma, can induce ferroptosis by enhancing DMT1 [32]. Another small molecule named MMRi62 is a more recently identified inducer that reportedly induces degradation of the ferritin heavy chain [33].

### Small molecules and drug inducers targeting lipid metabolism

**Fig. 2:**
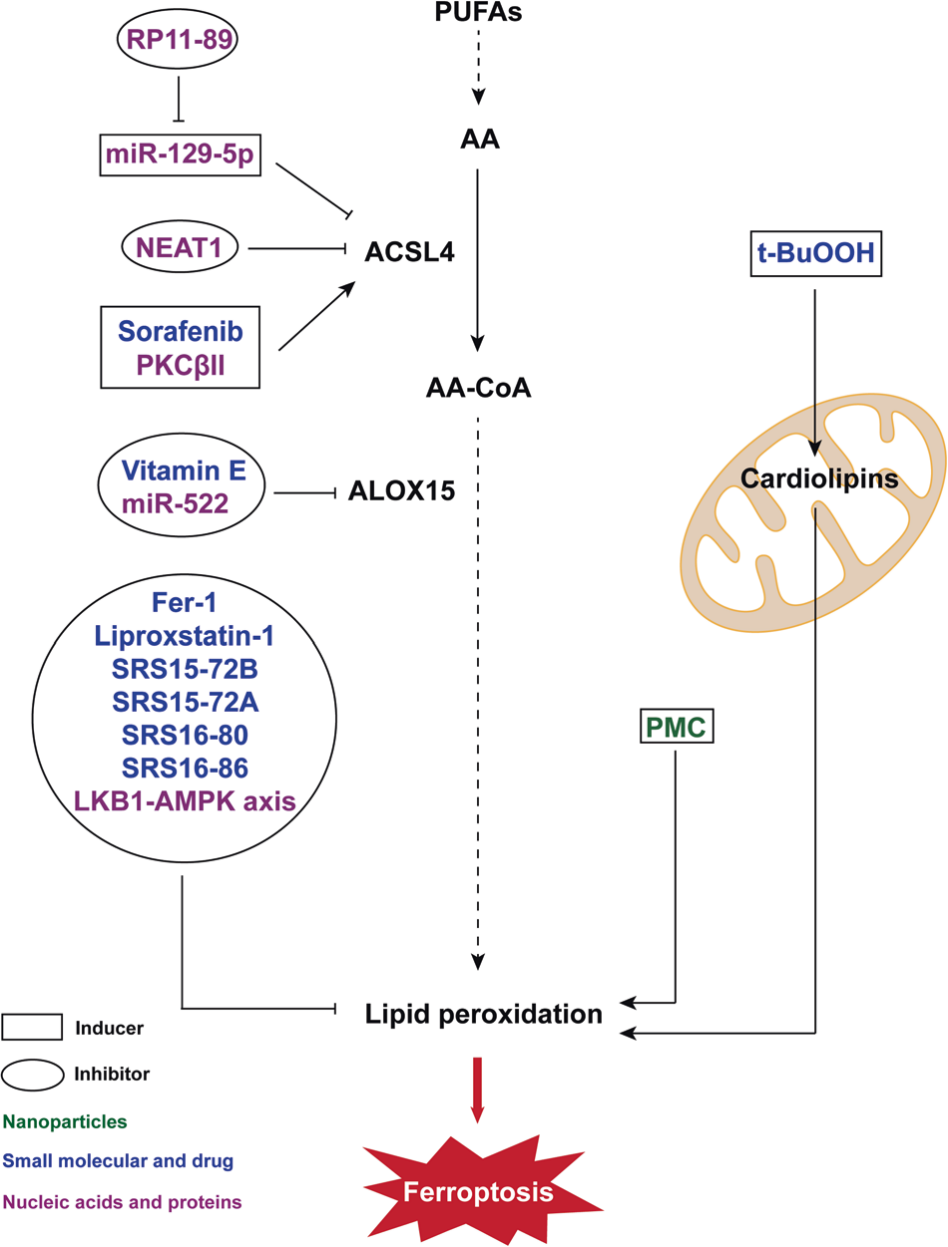
Substances that affect ferroptosis by regulating lipid metabolism. Lipid ROS accumulation caused by lipid peroxidation plays an important role in ferroptosis while antioxidants can prevent ferroptosis. Two of the important enzymes in lipid metabolism are ACSL4 and ALOX15, thus their regulators affect the process of ferroptosis. NEAT1 nuclear enriched transcript 1, Fer-1 ferrostatin-1, ACSL4 acyl-CoA synthetase long chain family member 4, ALOX15 arachidonate lipoxygenase 15, PUFAs polyunsaturated fatty acids, AA arachidonic acid, AA-CoA acyl-CoA, PMC photosynthetic microcapsule.

Ferroptosis can alternatively be induced by affecting lipid metabolism and directly producing lipid ROS (Fig. 2). In the context of the treatment of hepatocellular carcinoma, sorafenib, an FDA-certified antitumor drug, can induce ferroptosis in the presence of ACSL4 [34]. Particularly, the addition of sorafenib directly affects the metabolic pathway of lipid ROS production in cells. Another inducer that directly affects lipid ROS levels is tertiary-butyl hydroperoxide (t-BuOOH), which can lead to oxidative stress, abnormal mitochondrial membrane potential and DNA damage when administered. However, the collapse of the mitochondrial membrane potential is not the root cause of cell death. As a more recently reported inducer of ferroptosis, t-BuOOH functions through the oxidation of cardiolipins, which can be reversed by the inhibitors of cardiolipin oxidation, such as XJB-5-131 and JP4-039 [35]. The cell death caused by t-BuOOH can also be saved by cell-cell contacts through the hippo pathway [36].

### Small molecules and drug inducers targeting GSH/GPX4 axis

**Fig. 3:**
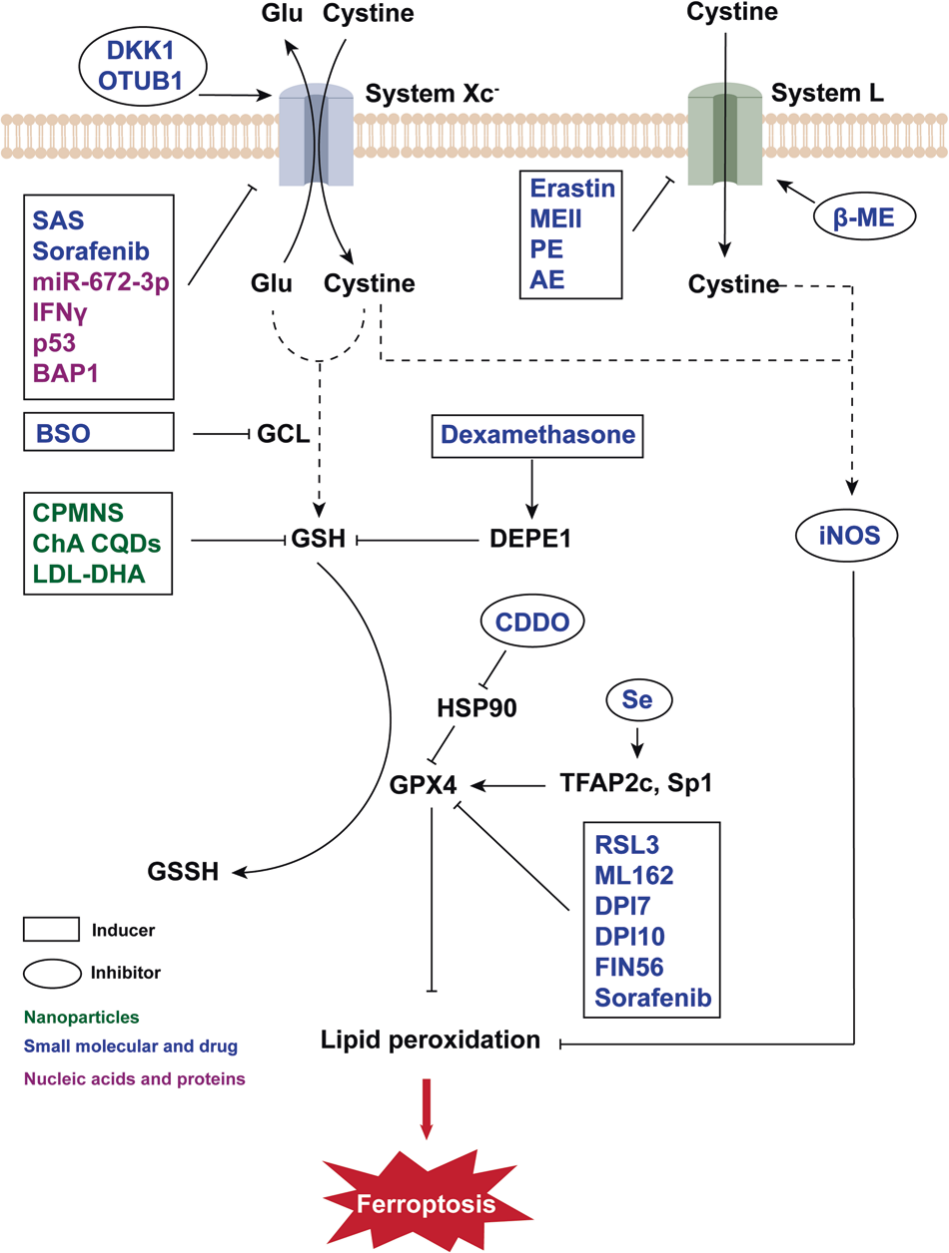
Regulators of ferroptosis through GSH/GPX4 axis. GSH is a crucial reductant, whose production involves amino acid transporters and a variety of enzymes, such as System Xc^−^ and GPX4, which are important targets for regulating ferroptosis. In addition, in specific cells like M1 tumor-associated macrophages, iNOS can catalyze the production of another reductant NO to inhibit ferroptosis. DKK1 dickkopf-1, OTUB1 ovarian tumor domain-containing ubiquitin aldehyde binding protein 1, Glu glutamate, MEII morpholine erastin II, PE piperazine erastin, AE aldehyde erastin, β-ME β-Mercaptoethanol, SAS sulfasalazine, BAP1 BRCA1-associated protein 1, BSO buthionine sulfoximine, ChA CQDs chlorogenic acid carbon quantum dots, LDL-DHA low-density lipoprotein-docosahexaenoic acid nanoparticles, GCL glutamate–cysteine ligase, GSH glutathione, DEPE1 dipeptidase-1, GPX4 glutathione peroxidase 4, CDDO bardoxolone, HSP90 heat shock protein 90, Se selenium, TFAP2c transcription factor activating protein 2 gamma, Sp1 specificity protein 1, iNOS inducible nitric oxide synthase, RSL3 RAS synthetic lethal 3, FIN56 ferroptosis inducing 56.

The production and oxidation process of GSH has multiple links that can be regulated (Fig. 3). RSL3 was discovered together with RSL5, though they function differently. RSL3 targets enzymes with nucleophilic active sites, acting on GPX4 to induce ferroptosis [37]. Other compounds, such as ML162, DPI7 and DPI10, have action modes similar to RSL3 [38]. Moreover, another compound called FIN56 can promote the degradation of GPX4 [23]. As discussed above, GCL is an important enzyme involved in the production of GSH, inhibition of which causes ferroptosis. As an example, buthionine sulfoximine (BSO) inhibits GCL, thus reducing the level of GSH in cells [39].

Some inducers have an ability to influence more than one target during these processes, among which the most widely used being erastin. In addition to blocking VDACs, erastin also affects the formation and oxidation process of GSH. System L is an amino acid transporter that differs from System Xc^−^ in the light chain, consisting of SLC7A5 and SLC3A2 [40]. Erastin combined with SLC7A5 leads to a decrease in the activity of System L. This process does not induce ferroptosis directly, but it has an impact on the vitality of System Xc^−^ and the cystine intake of cells [1]. In addition, erastin can deplete GSH and cause degradation of GPX4 [37, 41]. Some analogs of erastin, such as MEII, PE, and AE, have similar functions. Compared with analogs that do not lead to cell death, they have a better effect on the consumption of GSH [37]. Whether these analogs can bind VDACs and inhibit System Xc^−^ needs further research, but it is known that their lethal effect has been achieved by consuming GSH.

In addition to erastin and its analogs, a plurality of drugs are also known to block System Xc^−^. Sulfasalazine (SAS), certified by the FDA, inhibits System Xc^−^ and shows antitumor effects [42]. Sorafenib indirectly blocks System Xc^−^ by blocking its upstream regulator and also inhibits GPX4 indirectly [43–45].

### Small molecules and drug inducers targeting FSP1/CoQ related pathway

**Fig. 4:**
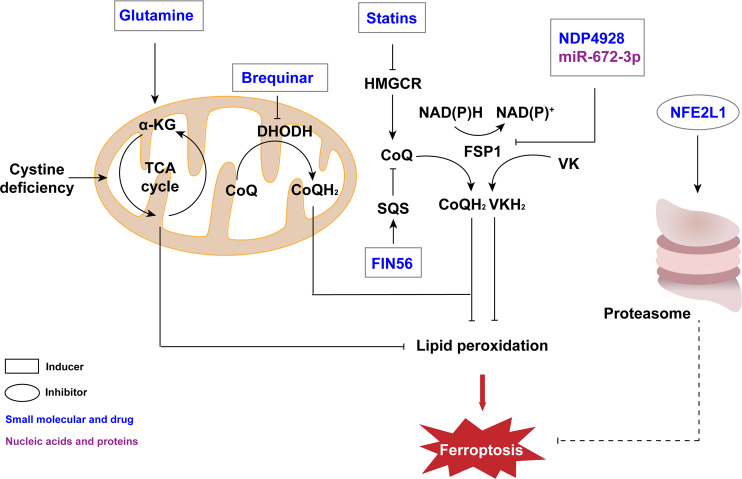
FSP1/CoQ and other metabolism pathways involved in ferroptosis. FSP1 can catalyze the transition from VK to VKH_2_ and CoQ to CoQH_2_ also catalyzed by DHODH to affect the process of ferroptosis. Thus, regulators targeting these two proteins such as miR-672-3p and brequinar can regulate ferroptosis. In addition, TCA cycle promoted by glutaminolysis can contribute to lipid peroxidation in the case of cystine deficiency to inhibit ferroptosis. Moreover, the proteasome regulates ferroptosis in a temporarily undefined manner. α-KG α-ketoglutarate dehydrogenase complex, TCA cycle tricarboxylic acid cycle, DHODH dihydroorotate dehydrogenase, CoQ coenzyme Q, HMGCR 3-hydroxy-3-methylglutaryl-CoA reductase, SQS squalene synthase, FIN56 ferroptosis inducing 56, FSP1 ferroptosis suppressor protein 1, NAD(P)H nicotinamide adenine dinucleotide phosphate, VK vitamin K, NFE2L1 nuclear factor erythroid-2, like-1.

NDP4928 is a small molecule that is more of an enhancer of ferroptosis than an inducer. It has been reported that NDP4928 shows weak cytotoxicity when alone, but its cytotoxicity could be greatly enhanced when cotreated with RSL3. Additionally, when cotreated with BSO, the cytotoxicity of NDP4928 was also enhanced. It is known that FSP1 is a target of NDP4928, and this compound binds and inhibits FSP1 to enhance ferroptosis induced by GSH inhibition [46]. FIN56 is another inducer with multiple effects in addition to erastin. FIN56 induces ferroptosis not only via degradation of GPX4 but also by binding with squalene synthase (SQS) and depleting CoQ [47]. Statins are widely used in the treatment of many diseases. Some of them have been certified by the FDA. Statins has been formulated into therapeutic nanoparticles [48] which block 3-hydroxy-3-methylglutaryl-CoA reductase (HMGCR) in the mevalonate pathway. As CoQ was reduced, ferroptosis occurred subsequently [49] (Fig. 4).

### Small molecules and drug inducers targeting other pathways

Although most ferroptosis inducers work through the three ways mentioned above, there are some inducers affecting other targets (Fig. 4). As an example, DHODH can be inhibited by brequinar. Therefore, brequinar can inhibit tumor growth by inducing ferroptosis in tumor cells [24]. Dexamethasone, a widely used drug, increases cell sensitization to ferroptosis by upregulating a regulator of the GSH metabolism pathway called dipeptidase-1 (DPEP1) [50]. With the discovery of new induction targets, the action modes and types of inducers are expected more diversified in due course.

### Nanoparticles inducing ferroptosis

Some nanoparticles with special structures and function can induce ferroptosis (Table 1). These nanoparticles have commonalities that can cause changes in iron level or ROS level (Figs. 1, 2).

One of the representative species related to iron level is Cornell prime dots (C’ dots). It has been reported that Cells are more sensitive to C’ dots when cultured in a medium free of amino acids. Multiple tumor cell lines can be induced to ferroptosis by C’ dots under starvation, and the death of cells also had a wave-like pattern. The mechanism of this phenomenon is hypothetically because of the ability of C’ dots to load iron since it is a silica nanoparticle [8]. (UPDA-PEG)@Fe^2+/3+^ nanoparticles designed by Chen et al. function in a similar way as C’ dots [51]. It can chelate large number of iron ions and load them into cells. Superparamagnetic iron oxide nanoparticles (SPION) can also increase intracellular iron level due to its own degradation [52]. Besides ferroptosis, SPION has been reported to induce apoptosis [53], necrosis [54] and autophagy-mediated cell death in different cell lines [55]. In addition to C’ dots and SPION, Xu et al. found that WS_2_ and MoS_2_ can induce ferroptosis by affecting lysosomes, resulting in Fe^2+^ leakage into cytoplasm [56]. These nanoparticles cause Fenton reaction in cells, GA-Fe(II), a type of ultrasmall gallic acid-ferrous nanocomplexes, is similar to them [57].

Another category of nanoparticles that induces ferroptosis does not affect ROS level through iron ions, but in other ways such as GSH consumption and Fenton-like reactions (Fig. 3). As an example, hybrid CoMoO4-phosphomolybdic acid nanosheets (CPMNS) as well as low-density lipoprotein (LDL)-docosahexaenoic acid (DHA) nanoparticles can induce ferroptosis by eliminating GSH and promoting the production of lipid ROS [58, 59]. A recently study showed that a nanozyme, ChA CQDs, which was prepared from chlorogenic acid (ChA) and based on carbon quantum dots (CQDs), was capable of oxidizing GSH and affecting lipid repair systems [60]. In addition, besides iron ion, some other metal ions can lead to Fenton-like reaction (Fig. 1). Oxidation of Co^2+^ can cause depletion of GSH and accumulation of lipid ROS [61]. CPMNS found by Wu et al. induces ferroptosis through accelerated Mo(V)-Mo(VI) transition [58]. Further, zinc ions released by zinc oxide nanoparticles can also achieve lethal effects [62]. Apart from these, producing ROS directly is another viable strategy considered by researchers for ferroptosis inducers (Fig. 2). As an example, photosynthetic microcapsule (PMC) is a newly engineered ferroptosis inducer designed by Jiang et al., which takes the advantages of the oxygen production capacity of cyanobacteria to produce ROS [63].

Combining nanoparticles and small molecular inducers or drugs has been shown to achieve a better effect. For example, choosing nanoparticles that have specific targets could achieve accurate delivery of ferroptosis inducers [45, 48, 64]. Song et al. designed intracellular-acidity-activatable dynamic nanoparticles, carrying RSL3 to induce ferroptosis of tumor cells [65]. Recently, Zheng et al. designed a ferroptosis strategy which combined nanoparticles with ultrasound to achieve better effect in the treatment of tumor [66].

### Nucleic acids and proteins with inducing effects

Noncoding RNAs, including microRNAs, play a regulatory role in multiple cellular processes and regulate ferroptosis with multiple targets [67]. The miR-672-3p is a microRNA that is differentially expressed after spinal cord injury (SCI). The expression of miR-672-3p was significantly increased after SCI and induced ferroptosis by inhibiting FSP1 [68]. As another example, Zhang et al. recently discovered that nuclear paraspeckle assembly transcript 1 (NEAT1) induces ferroptosis by competitively binding to miR-362-3p and promoting the expression of myo-inositol oxygenase. However, it is worth noting here that previous studies have proved that another long non-coding RNA nuclear enriched transcript 1, also called NEAT1 for short, can inhibit the expression of ACSL4 and the occurrence of ferroptosis, which is contrary to the former [69, 70].

Some proteins have been reported to regulate ferroptosis. In the immune response caused by cancer or immunotherapy, interferon gamma (IFNγ) secreted by CD8^+^ T cells downregulates two subunits of System Xc^−^ [71]. SLC7A11, one of the subunits of System Xc^−^, cannot only be repressed by miR-672-3p but also downregulated by p53 and BRCA1-associated protein 1 (BAP1) [72–74] (Fig. 3).

The tricarboxylic acid (TCA) cycle is an important metabolic process (Fig. 4). This process involves multiple reactions and enzymes. The glutaminolysis pathway is related to TCA cycle. It has been reported that ferroptosis induced by cystine deficiency can be inhibited by knockdown of the α-ketoglutarate dehydrogenase complex, acetyl-CoA carboxylase and fumarase [75, 76].

PKCβII belongs to the protein kinase C (PKC) family (Fig. 2). It could phosphorylate ACSL4 and amplify the level of lipid peroxidation, eventually causing ferroptosis [77]. In addition, raising the level of iron ions can also induce ferroptosis directly. As an example, nuclear receptor coactivator 4 (NCOA4) regulates ferritin, an iron storage protein. It has been reported to promote the degradation of ferritin in a lysosome-dependent manner [78].

## Ferroptosis inhibitors

Compared to ferroptosis inducers, the number of inhibitors is not as abundant, and small molecule compounds account for only a small portion (Table 2). The development of inducers is of great value in tumor therapy. Similarly, ferroptosis inhibitors can be applied in tumor immunotherapy and other diseases such as stroke [9]. Like inducers, they can also be categorized by their modes of action.

**Table 2:** Examples of ferroptosis inhibitors.

Type	Induction mode	Targets	Names	Reference
Small molecular and drug	Reduce iron levels	Iron ions	CPX, DFO, DFP, DFX	[1, 79–81]
	Reduce lipid peroxides	Lipid	Fer-1, liproxstatin-1 SRS15-72B, SRS15-72A, SRS16-80, SRS16-86, VKH_2_	[1, 7, 84–86]
		ACSL4	Nuclear enriched transcript 1(NEAT1)	[70]
		ALOX15	α-tocopherol (Vitamin E)	[115]
	Effects on the GSH/GPX4 axis	Cystine	β-ME	[1]
		TFAP2c, Sp1	Se	[9]
		HSP90	CDDO	[41]
Nucleic acids and proteins	Reduce iron level	Ferritin	Prominin2	[89]
		ALOX15	miR-522	[94]
		miR-129-5p	RP11-89	[93]
	Reduce lipid peroxides	ACC1	LKB1-AMPK axis	[96]
		/	iNOS	[97]
	Effects on the GSH/GPX4 axis	System Xc^−^	DKK1, OTUB1	[101, 104]
	Regulate the proteasomal activity	/	NFE2L1	[27]

### Small molecular inhibitors that reduce iron levels

Elimination of excessive iron ions is a direct way to inhibit ferroptosis (Fig. 1). For example, ciclopirox olamine (CPX) has been reported as an iron chelator that also has broad-spectrum antifungal and antibacterial ability [1]. In addition to inhibiting ferroptosis by chelating iron ions, the olamine salt CPX (CPX-O) has been proven to induce the degradation of ferritin in mice with polycystic kidney disease [79]. Deferoxamine (DFO) is another widely used iron chelator [1], which has been reportedly used to inhibit ferroptosis with a therapeutic effect on traumatic spinal cord injury [80]. Besides CPX and DFO, many other iron chelators have been reported, such as deferiprone (DFP) and deferasirox (DFX) [81].

### Small molecule inhibitors for reducing lipid peroxides

Ferrostatin-1 (Fer-1) was identified as a ferroptosis inhibitor by Dixon et al. in 2012, which was proved to inhibit RSL3 or erastin induced ferroptosis in HT-1080 cells. It has been shown that Fer-1 functions by inhibiting lipid peroxidation because of the primary aromatic amine [1]. In addition, Fer-1 can save ferroptosis caused by hyperactive p53. As mentioned earlier, in the regulation of ferroptosis, p53 is an important protein which regulates System Xc- and can promote embryonic lethality by inducing ferroptosis when it is activated [72, 82] (Fig. 2). Apart from inhibiting ferroptosis induced by RSL3 or erastin, there are studies show that Fer-1 can increase the GSH level to inhibit ferroptosis in oligodendrocytes [83].

Another commonly used effective lipid antioxidant is liproxstatin-1. Like Fer-1, it can inhibit many inhibitors including erastin. Additionally, it can protect cells from ferroptosis in the absence of GPX4. Because of the above effects, liproxstatin-1 plays a protective role in many disease models [84]. In addition, ɑ-tocopherol (Vitamin E) and analogs of Fer-1, such as SRS15-72B, SRS15-72A, SRS16-80 and SRS16-86, have similar functions albeit different effects and stabilities [7, 84, 85]. Further, it has been demonstrated in a recent report that vitamin K (VK) can turn into its corresponding hydroquinone (VKH_2_), and this process can be catalyzed by FSP1 [86]. VKH_2_, the fully reduced forms of VK, has the ability to inhibit lipid oxidation and functions in the inhibition of ferroptosis [87].

### Small molecular inhibitors affecting GSH/GPX4 axis

It is known that β-Mercaptoethanol (β-ME) inhibits ferroptosis when System Xc^−^ is blocked in the process of helping cells uptake cystine [1]. In this process, β-ME reacts with cystine and forms a mixed disulfide. The mixed disulfide is transferred into cells via System L and produces cystine rapidly [88].

Increasing GPX4 expression can in turn inhibit ferroptosis (Fig. 3). This process can be regulated by selenium (Se) through the activation of the transcription factors TFAP2c (transcription factor activating protein 2 gamma) and Sp1 (specificity protein 1), which can be inhibited by transcriptional inhibitors. Moreover, it has been reported that cells with low GPX4 levels cannot be protected by Se [9]. Chaperone-mediated autophagy can promote degradation of GPX4. 2-Amino-5-chloro-N,3-dimethylbenzamide (bardoxolone, CDDO) is a triterpenoid compound that inhibits the molecular chaperone heat shock protein 90 (HSP90). CDDO has been proven to inhibit GPX4 degradation and protect cells from ferroptosis [41].

### Nucleic acids and proteins with inhibitory effects

Some proteins participate in the metabolism of iron (Fig. 1). For example, prominin2 can promote the formation of multivesicular bodies and exosomes. Ferritin is secreted from cells under the control of prominin2, thus increasing ferroptosis resistance [89].

There are many noncoding RNAs with inhibitory effect like LINC00336 [90]. RP11-89 is a long noncoding RNA that inhibits ferroptosis by regulating miR-129-5p, which has a ferroptosis inducing effect that mentioned above [91–93] (Fig. 2). In addition, miR-522 inhibits ferroptosis via another enzyme called arachidonate lipoxygenase 15 (ALOX15) [94].

The LKB1 (liver kinase B1)-AMPK (AMP-activated protein kinase) axis regulates ferroptosis by regulating the activity of acetyl-CoA carboxylase 1 (ACC1). It has been shown that ACC1 catalyzes the biosynthesis of fatty acids [95]. As a downstream substrate of AMPK, ACC1 is reportedly phosphorylated and inhibited when AMPK is activated by ferroptosis-related signals [96]. M1 tumor-associated macrophages have another metabolic pathway that inhibits the production of lipid ROS and are more insensitive to ferroptosis than the M2 subtype in the absence of GPX4 [97]. M1 subtype cells express inducible nitric oxide (NO) synthase (iNOS) and produce NO (Fig. 4). NO can eliminate lipid peroxidation and protect M1 subtype cells from ferroptosis [19].

Dickkopf-1 (DKK1) can inhibit the Wnt/β-catenin pathway and promote tumor development [98–100]. Recently, DKK1 was identified as a factor with a ferroptosis inhibitory effect. DKK1 protects tumor cells from ferroptosis and is indispensable for metastatic outgrowth. The inhibitory effect of DKK1 is achieved by increasing the expression of SLC7A11, a subunit of System Xc^−^ [101] (Fig. 3). Since DKK1 can inhibit the Wnt/β-catenin pathway, it indirectly reduces the activity of signal transducer and activator of transcription 3(STAT3) [102], thereby binding with the promoter region of SLC7A11 and inhibits its expression [103]. Additionally, it has been shown previously that SLC7A11 can be stabilized by the deubiquitinase OTUB1 (ovarian tumor domain-containing ubiquitin aldehyde binding protein 1). It has been reported that OTUB1 are highly expressed in human tumor cells, which are consequently protected from ferroptosis [104].

NFE2L1 (nuclear factor erythroid-2, like-1) is a transcription factor that has been shown to participate in the regulation of proteasomal activity [105] (Fig. 4). Ferroptosis inducers promote proteasomal activity and improve cell tolerance. Although the mechanism by which proteasomal activity protects cells from ferroptosis is still unclear, some evidence has been presented, suggesting that it may be related to ubiquitination [27].

## Application and prospects of ferroptosis

Multiple diseases can be treated with ferroptosis inducers or inhibitors. Ferroptosis has been applied in the curation of tumors, neurological diseases, organ injuries, etc. Many studies have provided evidence that ferroptosis can inhibit tumor growth. Ferroptosis inducers have been demonstrated to have cytotoxicity to multiple tumor cells both in vivo and in vitro [1, 8, 23]. In addition to the direct administration of ferroptosis agents, ferroptosis inducers can alternatively be carried by some targeted carriers, which showed better antitumor effects [48, 64]. As ferroptosis can be induced by some nanoparticles, such as C’ dots, modifying these nanoparticles is another effective way to induce ferroptosis specifically in tumor tissues. For example, varying sizes of Fe_3_O_4_ nanoparticles have shown varying abilities to induce ferroptosis [106]. In addition, regulating the sensitivity of immune cells to ferroptosis is a way to enhance the effect of tumor immunotherapy [19].

Some neurological diseases are also associated with ferroptosis. A recent study showed that a lipid-transporting glycoprotein named apolipoprotein E (ApoE), which is a major protein in Alzheimer’s disease [107], has the ability to protect cells from various ferroptosis inducers, including erastin and SAS. ApoE inhibits ferroptosis by reducing iron release from ferritin via activation of the PI3K/AKT pathway [108]. Furthermore, the nonheme iron exporter ferroportin1 was down-regulated in Alzheimer’s mouse model, which induces ferroptosis and causes neuronal death and memory impairment, and these symptoms can be ameliorated by ferroptosis inhibitors [109]. Intracerebral hemorrhage is a subtype of stroke, which is also related to ferroptosis. Blocking ferroptosis has a therapeutic effect in intracerebral hemorrhage [9], as well as the occurrence and progression of some other neurological diseases, such as Huntington’s disease, Parkinson’s disease and amyotrophic lateral sclerosis, which are all related to ferroptosis [23]. Recently, Yao et al. discovered that ferroptosis of retinal ganglion cell induced by pathologically high intraocular pressure is one of mechanisms of the glaucoma [110].

In addition to cancer and neurological disease, tuberculosis and autoimmune diseases may also be regulated by ferroptosis. Tuberculosis is induced by *Mycobacterium tuberculosis* (Mtb). Mtb infection causes necrosis of host cells and ferroptosis is shown to be one of the mechanisms involved [111]. A recent study revealed that neutrophils from patients with systemic lupus erythematosus suffer from ferroptosis. In patients’ neutrophils, the expression of GPX4 was inhibited [112].

The mechanism and relevant metabolic pathway of ferroptosis still need further study. With the continuous advancement of research, new questions also appear. For example, wave-like propagation is a special phenomenon that appears in ferroptosis [113]. GPX4 seems to be necessary for propagation, but the mechanism is still unclear [6]. There are also some potential inducers and inhibitors that are introduced with the study of the ferroptosis mechanism. For example, Torin 1 can inhibit the function of a central regulator of cell growth and proliferation named mTOR. Torin 1 has been shown to protect cells by reducing the amount of System Xc^−^ under glucose deprivation [114]. Whether Torin 1 can be applied as an inducer of ferroptosis needs more evidence. Moreover, there are some compounds whose functions may be two-fold. MG132 is a proteasome inhibitor. Treatment with MG132 increased the expression of System Xc^−^ since the deubiquitinase OTUB1 can stabilize System Xc^−^ in a proteasome-dependent manner [104, 114]. Thus, MG132 may be a potential ferroptosis inhibitor in this regard. However, another study showed that the expression of the proteasome was blocked by ferroptosis inducers. Promotion of proteasome expression is thus beneficial to cells under ferroptosis [27].

## Conclusions

In summary, ferroptosis is a form of programmed cell death with high application prospects but still needs further study. The development of inducers and inhibitors is crucial to research on ferroptosis. Continuous discovery of new ferroptosis targets and mechanisms lead to new therapeutic drugs and methods for various types of diseases.
