# Correction: Recent progress in ferroptosis: inducers and inhibitors

**DOI:** 10.1038/s41420-023-01431-z

**Published:** 2023-04-17

**Authors:** Yunxi Du, Zhong Guo

**Affiliations:** 1 Center for Biological Science and Technology, Guangdong Zhuhai-Macao Joint Biotech Laboratory, Advanced Institute of Natural Sciences, Beijing Normal University, Zhuhai, China; 2 Key Laboratory of Cell Proliferation and Regulation Biology of Ministry of Education, College of Life Sciences, Beijing Normal University, Beijing, China

**Keywords:** Cancer metabolism, Targeted therapies

Correction to: *Cell Death Discovery* 10.1038/s41420-022-01297-7, published online 29 December 2022

The original version of this article contained an error in the references. The authors state the following: Upon further review, we discovered that we cited a literature (Ref. 54, DOI: 10.2147/IJN.S13244, retraction on March, 2022) in our article, which was not retracted at the time of writing our manuscript in beginning of 2022. We deeply regret that we did not detect this error before submitting our manuscript. We apologize for any inconvenience this may have caused to you and the readers of Cell Death & Discovery. We understand the significance of accuracy in scientific publications and are taking immediate steps to rectify the error. As this literature only provides supplementary evidence for our viewpoint, we would like to request that it be deleted and our viewpoint supported by other literature (Ref. 53 in our article). The original article has been corrected.

